# A case report of giant malignant schwannoma of the sciatic nerve associated with neurofibromatosis-1: A CARE-compliant article

**DOI:** 10.1097/MD.0000000000036358

**Published:** 2023-11-24

**Authors:** Kemal Gokkus, Murat Saylik, Tayfun Birtay, Mehmet Sukru Sahin

**Affiliations:** a Baskent University, Alanya Research and Practice Hospital, Department of Orthopedics and Traumatology, Antalya, Turkey; b Department of Orthopaedic Surgery, Istinye University Medical Faculty, Topkapi Kampüsü, Maltepe Mah, Edirne Çirpici Yolu, İstanbul, Turkey; c Baskent University, Alanya Research and Practice Hospital, Department of Anesthesiology, Alanya/Antalya, Turkey.

**Keywords:** Malignant peripheral nerve sheath tumors, malignant schwannoma, neurofibromatosis-1, sciatic nerve, sciatica

## Abstract

**Rationale:**

Neurofibromatosis type 1 (NF1) is an autosomal dominant neurocutaneous syndrome that causes multiple central and peripheral nerve sheath tumors. People with NF1 have a 10% chance of developing malignant peripheral nerve sheath tumors (MPNSTs). Here we report a unique instance of a malignant schwannoma that has remained free of metastasis since its initial removal a decade ago. The malign schwannoma has been infrequently documented in the literature, and remarkably, no instances of such an extensive postoperative time without metastases have ever been described.

**Patient concerns:**

A 46-year-old male patient with NF had multiple neurofibromas in different parts of his body, underwent surgery about 10 years ago (2013), and was diagnosed histopathologically as MPNST.

**Diagnoses:**

He was admitted to our institution with a recurrent mass in the posterior third of the proximal thigh and severe pain radiating to the left lower extremity, which presented as sciatic pain (2021). A magnetic resonance imaging and fluorodeoxyglucose-positron emission tomography examination revealed that the tumor was likely malignant.

**Interventions:**

Surgical excision was performed.

**Outcome:**

A 10-year follow-up revealed no metastases or neurologic impairment.

**Lessons:**

When articles about benign schwannomas are placed in a separate category, little is written about NF-1-related malignant schwannomas of the sciatic nerve. MPNSTs are high-grade, aggressive sarcomas with a high risk of local recurrence (40%–65%) and metastasis to other body parts. Therefore, among the various benign peripheral nerve sheath tumors in NF-1 patients, the diagnosis of MPNST is crucial.

Orthopedic surgeons should be aware that neurofibromas in NF-1 have a significant risk of developing MPNSTs. This study reports the successful treatment of a giant malignant sciatic nerve schwannoma with a long follow-up period without metastasis.

## 1. Introduction

Neurofibromatosis-1 (NF-1) is commonly known as Von Recklinghausen disease. It occurs in approximately 1 in 2500 to 3000 people and has no gender preference.^[1]^

NF-1 is a multisystem disease with great individual heterogeneity. Diagnostic criteria include the presence of at least 2 of the following: a first-degree relative with NF-1, 6 or more café-au-lait spots, 2 or more neurofibromas or a plexiform neurofibroma, optic nerve glioma, bone dysplasia, axillary or inguinal freckles, and 2 or more Lisch nodules.^[1]^

NF-1 is an autosomal dominant neurocutaneous syndrome that causes multiple central and peripheral nerve sheath tumors. Most of these tumors are harmless.

Neurofibromas.^[2,3]^

However, people with NF-1 have a 10% chance of developing malignant peripheral nerve sheath tumors (MPNST), which often grow within a neurofibroma.^[4,5]^

The vast majority of MPNSTs are aggressive, high-grade sarcomas with a significant risk of local recurrence (40%–65%) and distant metastasis. The lung is the most common site of metastasis, followed by bone, brain, and liver.^[1]^ This case report is noteworthy because it reports the successful excision of such a large tumor with a long follow-up time without metastasis of a malignant schwannoma of the sciatic nerve.

## 2. Case description

This case report complied with the Declaration of Helsinki and ethical approval was not required as the study was a case report. Written informed consent was obtained from the patient. This case report was in accord with CARE guidelines.

A 46-year-old male patient with NF had multiple neurofibromas in various locations on his body, was operated on about 10 years ago (2013), and was diagnosed histopathologically as a malignant schwannoma. His mother and brother were diagnosed with NF-1. Thus the family story was also fitted with NF.

**Figure 1.**
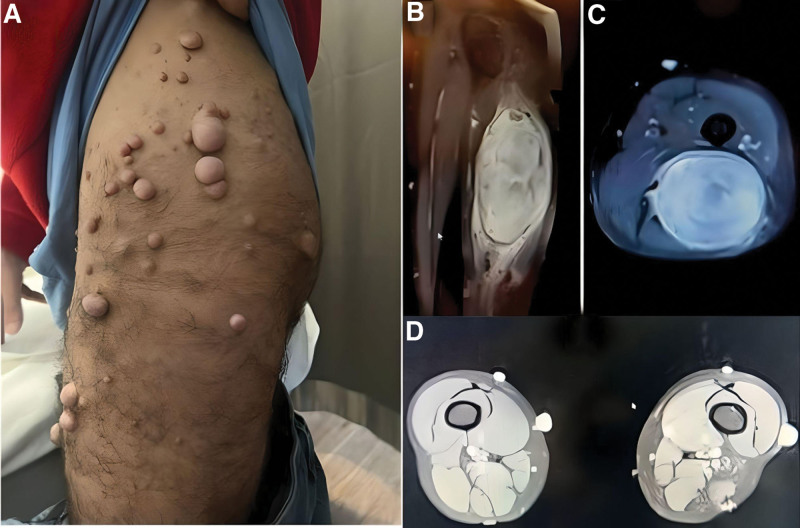
(A) Notice the multiple neurofibromas on the lateral aspect of the left thigh. (B) Contrast-enhanced MRI showed a 7.5*8.5*14.5 cm solid mass (schwannoma) extending along the sciatic nerve; it was mildly hyperintense on T1-weighted image. (C) Notice the tumor markedly contrasted and hyperintense on T2-weighted axial image on post-contrast sequence. (D) MRI follow-up, T1-weighted images, showed no recurrence. MRI = magnetic resonance imaging.

He was admitted to our institution with a recurrent mass in the posterior third of the proximal thigh and severe pain radiating to the left lower extremity, which presented as sciatic pain (2021). Physical examination revealed a partially mobile and painful mass in the posterior aspect of the thigh, a positive tunnel sign on the nerve, and a positive left plaque test. The patient systematic neurologic examination was routine and showed no neurologic deficits. The multiple soft pea-sized bumps on or under the skin (neurofibromas—see Figure 1A) and 6 or more cafe´-au-lait macules have been inspected.

**Figure 2.**
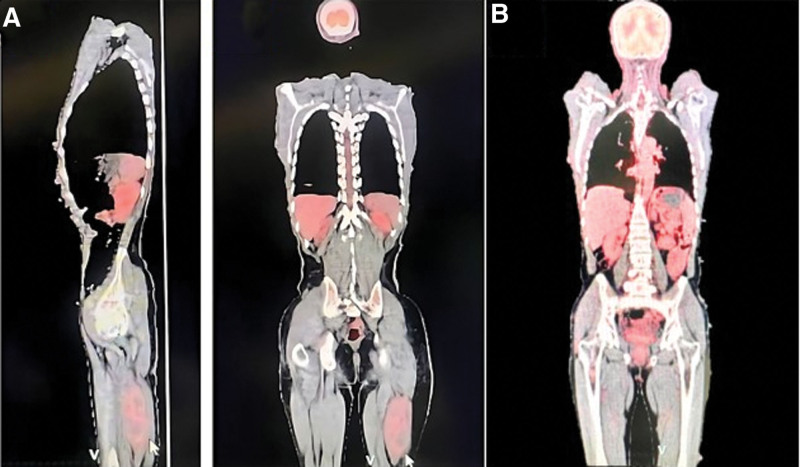
(A) FDG-PET scan showed a solid mass with enhanced metabolic activity in the left dorsal thigh and neurofibromas throughout the body under-skin. No metastases was noted. (B) After 10 yr follow up FDG-PET scan showed no metastasis. FDG-PET = fluorodeoxyglucose-positron emission tomography.

Contrast-enhanced magnetic resonance imaging (MRI) showed a 7.5*8.5*14.5 cm solid mass (schwannoma) extending along the sciatic nerve; it was mildly hyperintense on T1-weighted images (Fig. 1B), markedly hyperintense on T2-weighted images, and markedly contrasted on the post-contrast image (Fig. 1C). The presence of possible metastasis was investigated using fluorodeoxyglucose-positron emission tomography (FDG-PET), and there was increased activity of recurrent schwannoma in the left sciatic nerve and neurofibromas widely distributed over the body and under the skin; fortunately, no metastasis was detected (Fig. 2A). Our case was graded as grade 2 (SUV value according to the Musculoskeletal Tumor Society classification).

Surgical excision was decided, and before surgery, the patient was informed that a temporary or permanent neurological sensory or motor defect may occur. The patient is placed in a prone position. The posterior approach to the thigh was selected, the gap between the semitendinosus muscle and the biceps femoris muscle was identified, the biceps muscle was retracted laterally, and the semitendinosus muscle was retracted medially. The large mass overlying the sciatic nerve was exposed.

**Figure 3.**
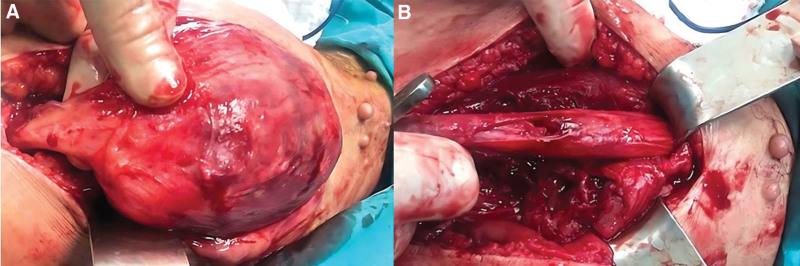
(A) Notice the appearance of the well-demarcated, solid, encapsulated tumor originating from the sciatic nerve sheath. Notice the typical subcutaneous neurofibromas on the upper side of the wound. (B) The intraoperative photograph shows the integrity of the sciatic nerve and the cloaca of the remaining base of the excised tumor over the nerve sheath.

A well-demarcated, solid, encapsulated tumor arose from the sciatic nerve sheath (Fig. 3A). The nerve sheath was transected, and the tumor was enucleated from the confluent nerve fascicles with the capsule. It was possible to remove the tumor entirely without damaging the fascicle structure of the sciatic nerve. The size of the mass was measured as 15 cm.

After the mass was removed, the integrity of the sciatic nerve was checked (Fig. 3B), and the nerve sheath was restored with 4-0 Vicryl by applying approximation sutures. The patient suffered from causalgia on the dorsum of the foot, indicating a sensory traction injury to the nerve; otherwise, he was neurologically intact postoperatively. Causalgia was controlled by oral administration of gabapentin. The patient has rejected adjuvant chemotherapy and radiotherapy for several reasons.

**Figure 4.**
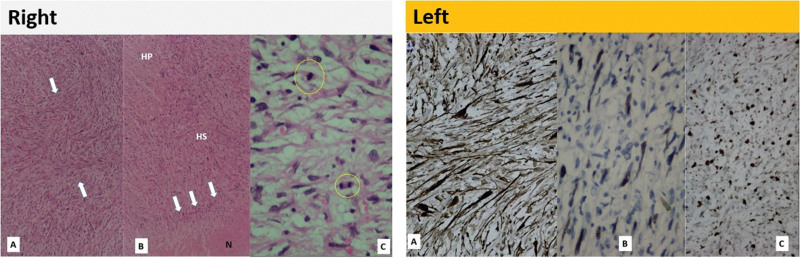
(Right). (A) Tumor tissue consists of wavy bundles of spindle-shaped cells (HE50x). (B) Hypocellular (HP) and hypercellular (HS) areas, plastics lines of cells (arrows) and foci of necrosis (N) are present (HE50x). (C) Cells with markedly hyperchromatic, pleomorphic nuclei, with atypical mitosis (within the yellow circle) (HE400x). (Left). (A) Immunohistochemical study, in spindle-shaped cells the cytoplasmic and nucleus S100 protein expression (DAB Chromogen 200X) (B) Nuclear Sox10 expression (DAN chromogen 400X) (C) Increased index of Ki67 (DAB Chromogen 200X).

Following the second surgical procedure, the excised mass underwent histopathological examination. On histopathologic examination, the tumor tissue exhibited spindle-shaped cell fascicles with undulating, comma-shaped hyperchromatic nuclei. The tissue revealed localized necrosis, and hypocellular and hypercellular areas were observed (see Fig. 4, right). The tumor cells exhibited a markedly increased mitotic pattern. Immunohistochemical analyses (see Fig. 4 left) revealed that the tumor cells expressed S-100 protein and nuclear Sox10 and had a higher Ki67 index. Desmin, CD34, and pan-cytokeratin yielded negative results. Based on these findings, the patient was diagnosed with a MPNST.

After a follow-up period of 10 years (since the first excision), the patient had no signs of metastasis on the FDG-PET scan (Fig. 2B). After the second surgery (2 years of follow-up), he had no local recurrence on MRI (Fig. 1D), causalgia gradually decreased, and the lower extremity was fully functional again. Analyze the timeline table to understand all events, episodes, and interventions sequentially (see table 1).

**Table 1:** A chronological table summarizing all events, episodes, and interventions in order.

Timeline—historical and current information from this episode of care organized as a timeline					
Yr	Family story	Primary diagnosis	First intervention	First histopathologic exam	First follow up and outcome
2013	His mother and brother were diagnosed as NF-1	NF-1	He was operated on about 10 yr ago (2013)	He was diagnosed with malignant schwannoma	Recurrence without metastasis (verified with PET–scan), 8 yr
		Diagnosis	Second İntervention	Second histopathologic exam	Second follow up
2021		Malignant schwannoma associated with NF-1	Second surgical excision has been performed	Malignant schwannoma	No recurrence without metastasis (verified with PET-scan), 2 yr
Total follow-up: a 10-yr follow-up revealed no metastases or neurologic impairment					

NF-1 = neurofibromatosis type 1, PET = positron emission tomography.

## 3. Discussion

This study describes a malignant schwannoma of the sciatic nerve associated with NF-1 that had not metastasized since the first excision 10 years ago.

Neurofibromas are the most common neoplasms associated with NF-1, but malignancies such as MPNST, non-lymphocytic leukemias, gliomas, pheochromocytomas, and gastrointestinal stromal tumors may also develop.^[3,4,6]^

An MPNST usually arises within a benign plexiform neurofibroma, and the lifetime probability of developing an MPNST in NF-1 patients is 10%.^[3,4]^

The prognosis of NF-1 patients with MPNST is poor, with a 5-year overall survival rate of 30%. Therefore, among the various benign peripheral nerve sheath tumors in NF-1 patients, the diagnosis of MPNST is crucial.^[3,7]^

If articles about benign schwannomas are put in a separate category, little is written about NF-1-related malignant schwannomas of the sciatic nerve. Only a few papers have been published on malignant schwannomas of the sciatic nerve associated with NF-1 ^[1,3,8,9]^ (see Table 2).

**Table 2:** Literature regarding the malignant schwannoma of the sciatic nerve associated with neurofibromatosis-1 (NF-1).

Literature regarding the malignant schwannoma of the sciatic nerve associated with NF-1					
The author name, journal and yr	Title	The association with NF	The number of patients and follow up yr	The size of tumor or intraoperative observation/note	The message of article
Topsakal et al^[9]^ Neurol Med Chir (Tokyo). 2001 Nov;41(11):551–5.	Malignant schwannoma of the sciatic nerve originating in a spinal plexiform neurofibroma associated with NF-1—case report.	Yes	2 mo the tumor recurred the patient died of an unknown cause.	25 × 30 cm	Despite complete macroscopic excision, the prognosis for MPNST associated with NF-1 is poor due to recurrence or metastasis.
Ghosh et al^[8]^ Cancer. 1973 Jan;31(1):184–90.	Malignant schwannoma. A clinicopathologic study.	Yes/some of patients	A study has identified 115 cases of malignant schwannoma in New York City between 1920 and 1970, the first of its kind in the United States. 10–15 yr	Local excision was performed on 58 patients with small tumors (<5 cm), resulting in a 5-yr survival rate of 79.3%. Fifteen patients underwent muscle group dissection, resulting in a 64.5% 5-yr survival rate. Tumor infiltration in the surrounding muscles was observed. Twenty patients underwent major amputation due to recurrent and extensive growth, resulting in a 50% cure rate over a 5-yr period. Out of the patients who underwent laparotomy or thoracotomy for tumor resection, only 27.4% survived beyond 5 yr. The total number of patients in this group was 22.	Out of 85 patients, 30 (26%) had malignant schwannomas that were linked to either multiple NF or plexiform neuroma. No significant gender or age preference was observed. The presence of plexiform neuroma or von Recklinghausen disease stigmata is associated with a negative prognosis. The co-occurrence of malignant schwannoma with plexiform neuroma or von Recklinghausen disease is indicative of a higher malignancy and a negative prognosis. The lesions exhibit greater pleomorphism on a histological level. Of the patient group, 30 individuals exhibited von Recklinghausen disease and had a 5-yr survival rate of 30%, which is significantly lower than the overall group 5-yr survival rate of 65.7%.
Evans DG et al^[7]^ J Med Genet. 2002 May;39(5):311–4.	MPNSTs in NF-1.	Yes	After careful review of hospital notes, 15 of the 54 MPNST patients were confirmed as having NF-1.	Not applicable (Prevalence study)	The incidence of MPNST in individuals with NF-1 is considerably greater than previously anticipated, necessitating vigilant monitoring and a cautious approach to investigation.
Chew et al^[1]^ J Radiol Case Rep. 2020 Dec 31;14(12):1–13.	Malignant transformation in a sciatic plexiform neurofibroma in NF-1—imaging features that aid diagnosis.	Yes	One patient/not applicable	A patient with 2 neurofibromas, one of which is malignant, has undergone surgery to remove a sciatic plexiform tumor and a new lung mass.	The distinction between malignant and benign tumors remains difficult in clinical settings. Radiology is crucial in providing answers to clinical inquiries. MRI is the preferred method for characterizing and outlining the target lesion, while CT and PET-CT are valuable for staging disseminated disease. PET-CT is also useful in determining the optimal location for image-guided biopsy.
Ahlawat et al^[3]^ Skeletal Radiol. 2013 Sep;42(9):1317–22.	Schwannoma in NF-1: a pitfall for detecting malignancy by metabolic imaging.	Yes	One case/	Not noted/complete resection of each of these masses was performed, yielding a histologic diagnosis of MPNST for the proximal anterior thigh ma.	In conclusion, this case emphasizes an important limitation to consider when using metabolic PET imaging to detect MPNST in patients with NF-1. Schwannomas, although uncommon in NF-1, can manifest malignant-like PET characteristics. Although conventional anatomic MRI features may aid in distinguishing MPNSTs from benign peripheral tumors, their characteristics frequently overlap, making it difficult to definitively distinguish between the 2 using MRI alone. A patient with NF-1 exhibited clinical indications of malignancy and underwent FDG-PET, which revealed 2 peripheral tumors with elevated metabolic activity. One of the tumors was identified as an MPNST, while the other was diagnosed as a schwannoma. The anatomic and functional MRI characteristics of the 2 masses differed, unlike in PET imaging.

CT = computerized tomography, FDG-PET = fluorodeoxyglucose-positron emission tomography, MPNST = malignant peripheral nerve sheath tumors, MRI = magnetic resonance imaging, PET = positron emission tomography.

In these papers, Ghosh et al reported detailed clinicopathologic observations in a carefully selected group of 115 patients diagnosed with malignant schwannoma. They reported that 30 patients (26%) had multiple NF (Von Recklinghausen disease).^[8]^

Topsakal et al reported a case of malignant schwannoma of the sciatic nerve arising from a spinal plexiform neurofibroma associated with NF-1. They performed resection without neurologic damage to the sciatic nerve. The tumor was very large (25 × 30 cm), and after 2 months, the tumor recurred, after which the patient died of unknown causes.^[9]^

In contrast to this case, neither recurrence nor metastasis occurred in the case described in this article, and the patient has remained healthy.

Ahlawat et al described a patient with NF-1 who had 2 peripheral tumors with similar features on PET, both of which were suspicious for MPNST but had different features on MRI, one of which was later found to be MPNST and the second a schwannoma.^[3]^ They concluded that functional and metabolic MRI techniques may complement PET in distinguishing benign peripheral nerve tumors from MPNSTs in NF-1.^[3]^

Evans et al performed a population-based longitudinal study to assess the lifetime risk of developing MPNSTs in NF-1 patients.^[7]^ They reported that 21 NF-1 patients developed MPNST, corresponding to an annual incidence of 1.6 per 1000 and a lifetime risk of 8% to 13%.^[7]^

Chew et al^[1]^ reported in “Malignant transformation in a plexiform sciatic neurofibroma in neurofibromatosis type 1—imaging features to support the diagnosis” that a 41-year-old Asian man presented with low-grade fever and an unintentional weight loss of 5 kg over 5 to 6 months: MRI of the left thigh revealed an 11 × 11 × 32 cm heterogeneous soft tissue mass in the posterior compartment. The authors performed surgical excision of the tumor in the posterior compartment of the left thigh, and the patient remained neurologically intact, but they did not report the exact follow-up time, so we could not learn about the patient metastasis and survival. The authors acknowledge that “FDG PET is a promising tool to distinguish between benign and malignant neurofibromas, plays a role in staging, and directs biopsy to the highest tumor grade. Computed tomography is an excellent way to delineate the extent of the tumor, detect bony involvement, and perform preoperative planning.”^[1]^

In the current report, PET has already been used to differentiate between benign and malignant neurofibromas and for possible tumor staging.

Sasaki et al investigated the effects of intraoperative monitoring of motor evoked potential (MEP) during surgical enucleation of peripheral nerve schwannoma on the prediction of nerve damage.^[10]^ They argue that MEP alone could not predict postoperative transient sensory or motor deficits after enucleation of a schwannoma.^[10]^ MEP was also not used in the present case because schwannomas are often well-encapsulated and displace nerve fibers as they develop. It is believed that enucleation can be performed without postoperative neurologic deficits.^[10]^

The malign schwannoma has been infrequently documented in the literature, and remarkably, no instances of such an extensive postoperative time without metastases have ever been described.

Clinicians are encouraged by the study findings that complete surgical excision of the tumor may spare the extremities from neurological deficits and metastases for an extended period of time.

In conclusion, orthopedic surgeons and neurosurgeons should be aware of this phenomenon. Contrast-enhanced MRI, PET, and functional and metabolic MRI techniques may help differentiate benign peripheral nerve tumors from MPNSTs in NF-1. Careful dissection of the tumor within the epineurium offers the possibility of excision without injuring the sciatic nerve. In this report, we report the successful treatment of such a large tumor with a long follow-up time without metastasis of a malignant sciatic nerve schwannoma.

## 4. Limitations of study

The main limitations of a case report refer to the limited possibility of generalizing the validity of the study and the impossibility of establishing a cause-effect relationship.

## Acknowledgements

The authors thank the patient for his participation and her agreement to publication of the report.

## Author contributions

**Conceptualization:** Kemal Gokkus, Murat Saylik, Tayfun Birtay, Mehmet Sukru Sahin.

**Data curation:** Kemal Gokkus, Murat Saylik, Tayfun Birtay.

**Formal analysis:** Kemal Gokkus, Tayfun Birtay.

**Investigation:** Murat Saylik.

**Methodology:** Kemal Gokkus, Murat Saylik, Mehmet Sukru Sahin.

**Supervision:** Kemal Gokkus, Murat Saylik.

**Validation:** Tayfun Birtay.

**Writing – original draft:** Kemal Gokkus.

**Writing – review & editing:** Kemal Gokkus, Murat Saylik, Mehmet Sukru Sahin.
